# Laparoscopic cholecystectomy during pregnancy: A case series

**DOI:** 10.4103/0972-9941.40991

**Published:** 2008

**Authors:** Hadi Ahmadi Amoli, Hassan Tavakoli, Ali Yaghoubi Notash, Maryam Sajad Far, Patricia Khashayar

**Affiliations:** Department of Surgery, Tehran University of Medical Science, Tehran, Iran

**Keywords:** Cholecystectomy, laparoscopy, pregnancy

## Abstract

This study was conducted to evaluate the safety of laparoscopic cholecystectomy (LC) during pregnancy. Patients who underwent LC were selected from several hospital databases, only six were performed during pregnancy. In this series, one of the two patients who had LC in the first trimester underwent elective termination of pregnancy while the other one gave birth to a term child normally. Half of the four who had the second trimester LC had normal deliveries at term whereas for the other two cesarean section was performed. None of our patients underwent LC in the third trimester. The findings of the present study suggest LC to be a safe procedure performed during the first and second trimester of pregnancy.

## INTRODUCTION

After appendicitis, biliary tract disease is the second most common general surgical condition encountered in obstetrical patients.[1] Gallbladder problems in pregnancy are usually due to the changes in hormone levels, especially higher levels of progesterone.[2] Gallstones are actually quite common in pregnancy and have been known to resolve after delivery, so even in the presence of stones, a wait and see approach would be the choice.[3] Some surgeons advocate an aggressive surgical approach in the management of gallbladder disease, while others favor medical management. Most agree cholecystectomy should be performed if conservative management fails, either during the same admission or in patients whose symptoms recur after resumption of oral intake, in the same trimester.[4] Although pregnancy was once considered an absolute contraindication to the performance of laparoscopic procedures, many recent case reports have shown favorable outcomes.[5,6]

Laparoscopic cholecystectomy in the pregnant patient is not yet broadly accepted; concerns have been for fetal wastage, effects of carbon dioxide (CO_2_) on the developing fetus and long-term sequel during childhood development.[7] Many believe the best time to do surgery is the second trimester; because in this period, the risk of miscarriage due to surgery is low and the uterus is usually small enough not to interfere with the laparoscopic approach. However it is still a controversial issue.[5,8–10]

## MATERIALS AND METHODS

The report documents six cases of laparoscopic cholecystectomy performed during pregnancy. After approval of ethic committee, the databases of three hospitals (Sina Hospital, Arad Hospital and Imam Khomeini Hospital) were reviewed for any pregnant patient who had undergone laparoscopic cholecystectomy during the interval of six years from 2000 till 2006. Our population consisted of six healthy pregnant women at 17, 12, 30, 13, 15 and 6 weeks of gestation. The dataset is outlined in Table 1. An informed consent for the laparoscopy was obtained from all the patients.

**Table 1:** Information on the target population

P,G	Age	GA (weeks)	Previous abdominal incision	CO_2_ pressure	Duration of procedure	Birth weight	Infant HC	Infant height
PIG4	23	17	No	12	75	3600	37	53
POG1	30	12	No	12	60	2825	34.5	48
PIG4	30	17	No	11	30	3600	36	51
PIG2	35	13	Yes	11	40	3100	34	52
PIG2	26	15	No	12	60	3500	36	51
PIG2	20	6	No	12	50	3300	33.5	50

The method used in our patients was the same in all the cases except the sixth. The laparoscopic procedure was carried out by the surgeons who had broad experience in operative laparoscopy. Under general anesthesia, the patient was placed in the Trendelburg position and the operating table was left tilted. The first trocar was entered using an open technique; Veress needle was not used to create a pneumoperitoneum. Pneumoperitoneum was created by insufflation with carbon dioxide (CO_2_) at an average intraabdominal pressure of 12 mmHg. Capnography which is a recommended, routine procedure for calculating end tidal CO_2_ was not utilized in our study. Monitoring including pulse oximetry, heart rate and blood pressure were performed in the entire operation. Fetal heart rate was evaluated by ultrasound before and after the surgery. A transvaginal sonography was used in case monitoring was required during the operation. In the last case, the surgeon was not aware of the patient being pregnant so pneumoperitoneum was created by Veress needle and the trocars were inserted via a closed technique. No intraoperative cholangiography was done in this study.

Tocolytic was not administered in neither of the patients; however, it was recommended in case premature contractions occurred after the operation.

## CASES

*Case 1:* A 23-year-old woman (Gravida 4, Para 1) presented at her 17 weeks of gestation with recurrent biliary colic. Her medical history included no previous intraabdominal operation. Physical examination revealed normal vital sign. CBC and LFT were reported to be normal. Cholelithiasis and gallbladder wall thickening were reported in the abdominal ultrasound. During laparoscopy, pneumoperitoneum was maintained with carbon dioxide for an average intraabdominal pressure of 12 mmHg. The LC operation lasted 75 min. She had an uneventful pregnancy and delivered at 41-weeks gestation by caesarian section as she did not have any labor pain. The baby was healthy, weighing 3600 g and having APGAR score of 9 at 1 min. Both mother and child were reported to be healthy.

*Case 2:* A 30-year-old woman (Gravida 1, Para 0) was referred at 12-weeks gestation with acute cholecystitis. She gave no history of previous intraabdominal operation. Laboratory values were normal. Sludge was documented by abdominal ultrasound. Antibiotic therapy (Ceftriaxone) was first applied for the patient but as there was no change in her general condition after 24 h, the surgeon decided to perform LC. The intraabdominal pressure was maintained for an average of 12 mmHg. The procedure took 60 min. An elective caesarian section was performed at 39-weeks gestation because of the patient's own will. A term infant of 2825 g, having APGAR score 7 at the first min and 9 in the fifth min was born. The subsequent evaluation of the baby revealed no abnormality in growth and development.

*Case 3:* A 30-year-old woman (Gravida 4, Para 1) was referred at 17-week gestation with acute cholecystitis. She had no previous history of any intraabdominal procedure. White blood cell count was 18600; other laboratory values were normal. Abdominal ultrasound showed cholelithiasis and gall bladder inflammation. The intraabdominal pressure was maintained for an average of 11 mmHg. The procedure took 30 min. An apparently healthy baby weighing 3600 g was born at 40-weeks gestation uneventfully, with an APGAR score 9 at the first min.

*Case 4:* A 35-year-old woman (Gravida 2, Para 1) was referred at 13-week gestation because of recurrent biliary colic. Her medical history revealed a previous intraabdominal operation; 14 years ago she had undergone a cesarean section. Laboratory values were normal. Multiple stones in gall bladder as well as gallbladder wall thickening were documented by abdominal ultrasound. The operation took 40 min. An infant weighing 3100 g with a normal apgar was born via caesarian delivery at 40-week gestation. The infant's APGAR score was reported to be 8 at the first min.

*Case 5:* A 26-year-old woman (Gravida 2, Para 1) was referred at 15-weeks gestation because of biliary colic. Her medical history was pertinent for recurrent biliary colic attacks. She did not give any history of previous intraabdominal pressure. Laboratory values were normal. Several gall stones without any gallbladder wall thickening were reported in the abdominal ultrasound. The intraabdominal pressure was maintained at 12 mmHg. The operation lasted one hour. A term infant weighing 3500 g with an APGAR score 10 at the first min was born in a normal delivery.

*Case 6:* The patient was a 20-year-old female (Gravida 2, Para 1) referred for recurrent biliary colic. Laboratory values were normal. Multiple cholelithiasis and gallbladder wall thickening were reported in the abdominal ultrasound. She underwent a LC. The intraabdominal pressure was maintained at 12 mmHg. The procedure took about 50 min. An enlarged uterus was reported during the operation; as a result, a pregnancy test was carried out revealed her pregnancy. Additional tests confirmed she was at six weeks' gestation at the time of LC. A term infant of 3300 g with an APGAR score 9 at the first min was the result of this pregnancy.

## RESULTS

Successful laparoscopic surgery was performed in all six cases. All the patients' clinical manifestations were resigned after the operation; they were all discharged in the first 48 h following the operation. No fetal distress or demise occurred, nor were any tocolytics used. No complications including maternal-fetal death, fetal death, premature contraction requiring treatment, infant respiratory distress, macrosomia, preeclampsia, bradycardia (newborn) and maternal dehydration were reported during the pregnancy period. The resultant children had no evidence of developmental or physical abnormalities during the study period.

## DISCUSSION

Approximately 1 in 500 pregnancies is complicated by a non-obstetric surgical condition.[11] Appendicitis, cholecystitis and ileus constitute the major surgical conditions.

Clinical symptoms are nonspecific and physical examination may be difficult to perform due to the enlarged uterus, making a precise diagnosis problematic.[12]

Biliary tract disease is reported to represent the second most non obstetric surgical emergency during pregnancy.[13] It has been postulated that pregnancy is associated with an increased percentage of colic acid, increased cholesterol secretion, increased bile acid pool size, decreased enterohepatic circulation, decreased percentage of chenodeoxycholic acid and increased bile stasis.[8] Pregnancy was once an absolute contraindication for laparoscopy. Nowadays, in the absence of a prospective, randomized, controlled trial comparing laparoscopic cholecystectomy, open cholecystectomy and conservative medical management for pregnant patients with symptomatic cholelithiasis, retrospective case reports and series provide some insight into the relative benefits of each treatment modality. Many studies have demonstrated suboptimal clinical outcomes following conservative medical treatment in these patients. These studies believe maternal illness pose a greater threat to the fetus compared to surgery. They have shown readmission to be greater than 50% in these patients; moreover, spontaneous abortion or preterm labor was reported in 16% of them. Laparoscopic surgery is also associated with a lower incidence of premature delivery because of decreased uterine manipulation.[14–16]

As a result, many studies support that laparoscopic cholecystectomy can be safely performed during pregnancy. In a recent review of literature, no complications of surgery were reported.[1,6,13,17,18] Moreover, laparoscopy is also an excellent tool when the diagnosis in a pregnant patient is uncertain.

At present, the general contra-indications for laparoscopy include:[19]

Absolute contra-indications:

- Hypovolemic shock, massive bleeding or hemodynamic instability.
- Severe cardio respiratory disease.
- Uncontrolled coagulopathies.

Relative contra-indications:

- Peritonitis
- Portal hypertension
- Multiple previous procedures/extensive intraabdominal adhesions

The use of laparoscopy in early pregnancy has been recommended in many studies; according to these studies the enlarged uterus and relatively smaller abdominal cavity result in difficulties when performing these procedures in advanced gestation. The risk of penetration of the uterus on the introduction of the Veress needle and trocar has led to the recommendation for insertion the abovementioned device under sonographic control or the use of open technique.[18] An additional concern unique to the laparoscopic surgery is the possibility of high intraabdominal pressure, decreasing venous return and cardiac output, resulting in the reduction of utero-placental blood perfusion. The Trendelenburg position may also aggravate the low lung compliance caused by increasing intraabdominal pressure; furthermore the pneumoperitoneal related complications and injuries to abdominal organs followed by trocar insertion are frequently reported in this procedure. Trendelberg position and operating table left tilt to avoid caval compression can minimize the risk of this complication.[20–22]

Steinbrook *et al.* have reported similar cardiovascular effects and hemodynamic changes after CO_2_ pneumoperitoneum in pregnant and non pregnant patients. In another word, similar decrease in cardiac index, mean arterial pressure and SVR (systemic vascular resistance) is reported after CO_2_ insufflation in pregnant and non pregnant patients.[17]

It is assumed that maintaining end tidal CO_2_ pressure (PET CO_2_) around 32–34 mmHg prevents significant respiratory acidosis during laparoscopic surgery in pregnant patients and as a result, capnography would be adequate to guide ventilation during CO_2_ insufflation in this group of patients.[23] On the contrary, Cruz *et al.* reported maternal and fetal acidosis in pregnant ewes when PET CO_2_ was used to guide ventilation during CO_2_ insufflation.[24] Repeated ABG has not shown to have more advantages compared with simple capnography because it is shown that an average pneumoperitoneum pressure of 12 or lower is not accompanied by a significant increase in CO_2_ level in mother or fetus blood and so fetus acidosis would not be a harass.[23]

The fetus normally maintains a mild respiratory acidosis, which facilitates tissue - oxygen delivery by shifting the oxyhemoglobin dissociation curve to the right. It is possible that any increase in maternal CO_2_, for instance during CO_2_ pneumoperitoneum, may impair the exchange and as a result, worsens the fetal acidosis. Thus, several studies have suggested routine intraoperative fetal monitoring. On the other hand, some others suggest fetal heart rate evaluation by ultrasound before and after the surgery would be sufficient. It should be noted that transvaginal sonography must be used during the procedure because the signals from abdominal ultrasound would be lost during insufflation.[4,25] Another concern is that the type of anesthesia might affect the fetus. The majority of the studies have noted general anesthesia to be the anesthetic method of choice in these patients; however, regional anesthesia can be safely used during the first and early second trimester.[10,25]

There is no evidence to support the routine use of prophylactic tocolytics; however, they have been administered when premature contractions developed after LC.[6,10]

In addition, using different radiologic techniques in order to confirm the diagnosis of biliary disease and intraoperative cholangiography during the pregnancy is not absolutely contraindicated and could be performed using a shield if necessary.[26,27] However, in our patients there was no need for performing CT-scan prior to the operation or intraoperative cholangiography for confirming the diagnosis was not required.

Morbidity ranges from 1 to 9% and CBD injuries from 0.2 to 0.7% and they both largely depend on the surgeon's experience. Conversion rates are from 1.8 to 7.8%.[28] Specific complications include hemorrhage, bile leaks, retained stones, wound infections and incisional hernias.[9,28–29]

In general, laparoscopic cholecystectomy has proven to have several advantages upon open surgery; some of the merits are as follows:[30]

- Shorter hospital stay
- Less time to resume normal duties
- Lower pain scores and less use of opioids
- Sooner return to normal diet
- Greater patient satisfaction

The key points associated with higher success rate in laparoscopic cholecystectomy during pregnancy:

- Experienced surgeon[28]
- Trendelberg and left tilted position[20–22]
- Insertion of the first trocar with great precussion especially in late pregnancy to avoid injury of gravid uterus[18]
- Pneumoperitoneum created with an average intraabdominal pressure of 10–12 mmHg[23]
- Adequate Mother monitoring (adequate management of anesthetic, adequate hydration of mother to reduce the likelihood of premature labor) and treating the contractions with tocolytics.[4]
- Conversion to open cholecystectomy should be performed if intraoperative conditions make continued laparoscopic surgery unsafe.[30–31]

## CONCLUSION

Laparoscopic surgery is now proving to be as safe as open surgery in pregnancy. No deleterious effects to either mothers or children have been reported throughout this study.
